# Book Reviews

**Published:** 1809-11

**Authors:** 


### CRITICAL ANALYSIS
### OF THE
### RECENT PUBLICATIONS
### ON THE
### DIFFERENT BRANCHES OF PHYSIC, SURGERY, AND MEDICAL PHILOSOPHY.

*Observations on the Hepatitis of India, and on the prevalent Use of Mercury in Diseases of this Country.* By William Saunders, M. D. F. R. S. &c. London, 1809.

*Examination of the Prejudices commonly entertained against Mercury as beneficially applied to most Hepatic Complaints, and to various other Forms of Disease, as well as to Syphilis.* By James Curry, M. D. F. A. S. &c. one of the Physicians to Guy's Hospital, and Lecturer on the Theory and Practice of Medicine.

It gives us much pleasure to find a subject of so much importance brought before the public, and by characters, who if not *cantare pares*, are at least *respondere parati*. The very general, and as it is by some called the indiscriminate use of mercury, is now a matter of universal notoriety: virgins who formerly would have been alarmed at the least taint in their breath, or the slightest possible injury to their teeth and gums, submit patiently to be salivated, and even sometimes salivate themselves. Let us enquire whether this is founded on so great a success in the remedy as to bear down all opposition, or to be considered among those fashions, which in medicine, as well as in other arts, is equally fluctuating and uncertain.

The writers we have now before us, are neither of them to be considered in the light of mere controversialists. Though it might not be fair to make a comparative view of the two, when we reflect on the lengthened experience of one of them; yet it must be admitted, that if this has already acquired a well earned reputation, the other may be not less solicitous to establish a character which very early attracted public notice. Thus neither can be considered as writing merely to acquire popular fame, nor with any other bias than those prepossessions, of which no author or practitioner can entirely divest himself.

Dr. Saunders's pamphlet is published as an Appendix to his Treatise on the Liver. This latter is so well known, (the present being the fourth edition) that we shall presume our readers well acquainted with it. The Appendix begins with some remarks on the Indian hepatitis, particularly in those points in which it may be more strikingly contrasted with the European diseases of the liver. The former is very properly considered, as arising from some local miasma, and constantly prevailing state of the atmosphere, which is is peculiarly destructive to strangers; and like all other strong marked epidemics, is found not only to influence more or less all other diseases, but not uncommonly to imitate them in its access and progress. Against this formidable disease, by the universal consent of the most able physicians, the immediate use of mercury is not only the best, but the only remedy that can be depended on; insomuch, that the business of the practitioner is almost confined to diligently exhibiting it in such forms as shall be the most likely to influence the functions of the different organs, before suppuration is formed in the liver.

Having admitted all this in favour of the early and vigorous exhibition of mercury in the Indian hepatitis, Dr. Saunders adds,

"It should also be remembered, that the liver diseases of this country are, generally speaking, much slower in their growth than those of India; and numerous instances might be adduced, in which an invalid labouring under the more chronic form of liver disease, who by prudent management and unirritating medicines (such as dilute solutions of saline purgatives, Cheltenham water, and the like,) might have retained for many years a share of health amply sufficient to render life desirable, had by a rash application of so active a remedy as mercury, been hurried to his grave in a few months."

So serious a remark, from one who having now become a consulting physician, must have large opportunities of seeing the result of others' practice, cannot be read without emotion.

We have been short in our remarks on this part of the Appendix, because the account of the Indian disease is professedly taken from the writings of others. In the "observations on the prevalent use of mercury in the diseases of this country," we are principally to expect information from a London practitioner. Dr. Saunders stands among the oldest of these, an advantage which, joined to his long habit of lecturing, has given him a more extensive acquaintance among medical men, and a larger means of learning the result of their individual practice, than any other physician can boast.

"My own experience, (says Dr. S.) has furnished me with the means of ascertaining, that even calomel, which is the most manageable preparation of mercury, cannot be employed with safety or success in a great variety of cases in which it has been recommended; it is, however, frequently preferred from having neither taste nor smell, and from its acting in a small bulk; but it ought seldom to be used by itself as an habitual purgative, or laxative, as its operation in that case is extremely uncertain; sometimes it produces mucous and bloody stools, accompanied with tenesmus and prolapsus ani, irritating and exhausting the power of the intestines, and laying the foundation of painful and dangerous strictures of the rectum. It is generally improper in dyspeptic complaints, especially especially in delicate and irritable habits; at other times, instead of acting on the bowels, it forcibly determines to the mouth, and produces all the inconvenience of a salivation, when not expected, or wished for. It enters into the composition of most of our worm medicines which are advertised for sale; and from the free and unskilful use of such in the hands of ignorant people, considerable debility, emaciation, and even convulsions in children, are induced."

The following passage is still more pointed, as it respects diseases, for some of which mercury is principally in use. The remarks seem also to arise from a sight of those subjects which, after the unsuccessful endeavours of others, have been consigned to the author.

"In hepatic diseases, where scrophulous tubercles are formed, and in other affections of the liver, where the structure has been destroyed by interstitial deposit, with adhesive inflammation obliterating organization; where the absorption of parts has taken place, diminishing the bulk of the organ; with a structure both spongy and loose; if the jaundice accompanying these appearances be fixed and unremitting, I have never seen any advantage from the use of mercury. On the contrary, I am persuaded, that life, which under all these unfavourable circumstances, might have been prolonged by other means, and by such as a well-regulated diet, and the moderate use of gentle, mild, opening medicines, has been shortened by mercury.

"And I have known many cases of confirmed dropsy, with diseased viscera, where the believers in the specific power of mercury have promised a cure, and where they have so committed themselves by their confidence in its power, as to have been disgraced by their temerity.

"In the morbid state of the kidney, and urinary passages, the schirrous state of the prostate gland, or similar diseased conditions of the uterus; in the ulcerated and cancerous state of these parts, the mercurial action shortens human life, notwithstanding ignorant and credulous practitioners are every day resorting to it, as the infallible specific of deeply seated diseases."

Some valuable practical remarks follow on the best mode of treating the common hepatic diseases of more northern climates, which it is shown often arise, if not from mere plethora, at least from partial congestion. The most judicious forms of exhibiting calomel as a purge are next stated, and also the mode in which that remedy produces its good effects. This part abounds in useful reflexions on the circumstances under which, not only calomel, but mercury in any form, will fail to excite its effects on the salivary glands. In many acute visceral diseases, it is proved to have little effect, and that even in the advanced stages of syphilis, its operation will often be tedious.

"Perhaps," continues our Author, "it is only under the most favourable state of fevers that mercury salivates, so that it is difficul difficult to determine how far the cure should be attributed to the use of it. We are assured that in India, it will seldom or ever salivate after an abscess is formed in the liver. The doses of calomel which have been giving to persons in the yellow fever is astonishing, without acting on the mouth or bowels:* such is the torpid state of the body in that disease, perhaps from some affection of the brain.

"It does not appear that, if we except the hepatic diseases of India, the mortality of the endemic fevers of other countries has been diminished by extending the use of mercury, except as uniting mercury with other purgatives.†

Such are the outlines of this Appendix. Some letters are added, of no small moment to the illustration of Dr. S's remarks, and of the operation of this important remedy. The first is from Mr. Paisley, formerly head surgeon at Madras. In this, the usual credit is given to mercury in the East India endemic, and in many other acute diseases, which are shown to be more connected with hepatic disorganization than was formerly suspected. But, it is absolutely necessary to impress our readers with one practical aphorism, strongly insisted on in this and a subsequent letter; namely, that though the constitutions of East Indians bear the free use of mercury so well, and with so much advantage in some acute diseases; though they are so insensible to its influence, as often to require relaxing remedies, and even bleeding to assist its effects and prevent the increase of inflammation; yet, in syphilis and other chronic diseases, "mercury given without bark will spread every ulcer, induce fibrillæ, and increase every symptom."

A Letter from Dr. Curry follows: It begins with remarking the danger of the exhibition of mercury in the acute hepatitis of England, in any other form than calomel. This remark is, however, confined to the hepatitis of northern regions, where the disease partakes more of the nature of simple inflammation. It may be thought, perhaps, that simple inflammation, whether seated in the liver or lungs, might be treated in the same way. But Doctor C. remarks, that in the liver it arises often from, or is increased by, the mere congestion of bile in the ducts. This is illustrated by inflammation of the mammæ, in which it is found necessary, not only by evacuant remedies, to lessen the inflammatory

* "The state and influence of the brain on fevers, is well explained by Dr. Clutterbuck, in his "Enquiry into the Seat and Nature of Fever;" a book which conveys much practical and useful observation.

† "Of twenty-seven recruits for the Royal Artillery, who arrived in Grenada in July, 1793, twenty-six were seized with the fever; and of those, twenty-one died before the middle of August ensuing, that is, in six weeks."—Dr. Chisholm had satisfied himself of the great virtues of mercury at least four months before, yet this is a mortality never exceeded in any fever.—Vide Dr. Chisholm's Essays, p. 97.

matory diatheses, but also to relieve the tubæ lactiferæ by drawing off the milk. This last process cannot be carried on in the same manner in the liver or its ducts. But for them, Dr. Curry assures us, that "calomel is specifically a cholagogue or evacuant of bile." For this reason, he wishes the calomel by no means to run through the bowels like a common cathartic, but to rest about the duodenum. "For this purpose," says Dr. C. "I have often been obliged to assist its relaxing power on the biliary ducts, by joining it with opium and antimonial powder, especially the former, which I give to the amount of a grain or more every six hours, or as often as the urgency of the pain renders necessary. Under this management, I have repeatedly found the urgent symptoms abate considerably, many hours before any alvine evacuation took place, and of course before the calomel could be said to act as a carthartic: in some cases too, where no evacuation followed, it became necessary to give cathartic medicines afterwards, in order to secure the relief which the calomel had procured, and to prevent the pain and dyspnœa from returning; which they were apt to do, if the liver was not emptied while under the relaxing influence of the calomel and opium. It not unfrequently happens, that the stomach partakes so much of the inflammatory condition of the liver, and becomes in consequence so extremely irritable, as to reject every thing by vomiting almost as soon as swallowed."

In these cases, even calomel united with opium would prove too irritating, and the best remedy was found to be hydrargyrus præcipitatus cinereus, which has answered every purpose. The local effect of calomel is further illustrated by the effect which Mr. Clare produced on the salivary glands, by rubbing two or three grains of it daily on the gums. In these cases salivation was quickly produced, but the constitution was not permanently affected.

As Dr. Curry's pamphlet will presently come before us, we shall be very short in our remarks on this Letter. Respecting the cholagogue virtues of calomel depending on its passing more slowly through the intestines, we confess ourselves unable to determine. But we have frequently witnessed the most common purgative producing, first, the effect of emptying the intestines of fæces, and afterwards of what is usually called bile. From emetics, we have seen similar effects after the contents of the stomach have been discharged. Nor are we perfectly satisfied with the analogy of the salivary glands: we conceive that almost any friction, continued for some time on the gums, might produce an increased secretion of those parts.

Under these circumstances, we are not prepared to admit all that Dr. Curry requires before he draws his inference. "Why a medicine, says he, possessing such a property, [of emulging the liver of its bile] should be especially serviceable in hepatitis, must, I think, be pretty obvious." It certainly might be obvious if we

 were were convinced that calomel in particular has such a property. But even then we have much to learn in order to ascertain the presence of hepatitis in many cases in which Dr. Curry has discovered it. Cases, having all the symptoms of peripneumonia, with pain in the *left* side of the thorax, and no symptoms present which clearly indicated hepatic affection, were at first considered as peripneumonic or pleuritic, and treated accordingly by local and general blood-letting, purging with infusion of senna, blisters to the part, and antimonial diaphoretics. No permanent advantage was derived, continues Dr. C. till the failure of these remedies led me to suspect that the liver was the primary seat of the complaint, and the remote thoracic pain only symptomatic.

"Upon this impression, I immediately put in practice the mode which I had before employed with success in obvious cases of hepatic inflammation,—of giving three or four grains of calomel every four or six hours, as the urgency of the symptoms required;—and with the effect of entirely removing the pain and difficulty of breathing in the course of the night."

We shall not take upon ourselves to determine whether the patient owed his relief to the previous evacuations, or the calomel, or to both. The exhibition of three or four grs. of calomel four or six times a-day, if it does not run off by stool, would be likely to induce some mercurial action, provided the acute disease did not so far occupy the constitution as to prevent irritation from any other cause: and the previous evacuations might have sufficiently reduced the inflammatory action to render the constitution susceptible of a new irritation. Our readers will consider this as mere matter of conjecture on our parts; but we conceive ourselves authorized in making such a conjecture, till Dr. Curry shall give us further information than the conciseness of this epistle will admit.

The third Letter is from Dr. Duncan, and contains many valuable facts. By this, the salutary effects of mercury in hepatitis and many other acute diseases are confirmed, but its deleterious consequences in other complaints are more strongly marked.

"The event, says Dr. D. however, is far different with respect to the syphilis, or supposed syphilis, and some disorders unconnected with the liver, where the use of mercury is productive of the most serious and irremediable mischief, and this in almost every instance where the patient is exposed to cold or wet, either during the mercurial course, or too soon after it has been laid aside. The number of soldiers who are obliged to be invalided on account of the incurable pains, swellings, and loss of power in their limbs, occasioned by mercury, would appear incredible to you; and it is melancholy to think how many officers lose their lives from the same cause, and under symptoms the most painful and lamentable, where the nose and great part of the face is destroyed, the bones rendered carious, and the surface of the body covered with ulcers. I have seen many fatal cases of this sort, and in none of them had mercury the least effect in removing or even mitigating the symptoms which were thus brought brought on from exposure to cold during the mercurial course, or after it was laid aside, and the cure supposed to be completed. Indeed I always thought that mercury aggravated the symptoms, and the patients themselves were sometimes sensible of this, and earnestly entreated that the medicine might be discontinued. Mercury was generally tried on the idea that these symptoms arose from some lurking remains of the venereal virus. But I was soon convinced that this was a mistake, and that the above symptoms, however nearly allied to those of the venereal disease, were occasioned by mercury itself."

Two very melancholy cases are related, as illustrations, not of the rare, but common consequences of the injudicious perseverance in mercury. The author is so much impressed with the subject, that he cannot check himself from adding other cases in illustration of all these dreadful maladies. So strongly had experience impressed him with the extreme danger attending mercury, that he became at last comparatively indifferent to venereal symptoms, satisfied that he had always a remedy for them, but none for the fatal effects of mercury. Venereal symptoms that occurred in the field, were therefore left to themselves till a fair opportunity arrived of giving mercury with safety.

"After a lapse of ten months, I could not trace any symptom of it beyond the groin. I did not find that the throat, bones, or skin, were affected; although, in the early stages of the disease, where mercury had been given, and the patient exposed to cold, all the worst symptoms soon made their appearance. Indeed, every case that I saw in India, tended more and more to convince me, that it is only in combination with mercury that syphilis produces all the worst symptoms, and that the progress of the disease when left to itself, is extremely slow. This opinion I adopted with great hesitation, but it was confirmed by facts which appeared otherwise inexplicable."

This is enough to show that mercury, however valuable a remedy, is not unattended with proportionate danger.

Such is Dr. Saunders's Appendix; and if, as we believe, it makes the only difference between this and the former edition, we trust he will so far consider the purchasers of the last, as to direct this addition to be sold separately.

---

An Advertisement prefixed to Dr. Curry's Pamphlet informs us, that it was drawn up more than a Year ago, as an Introduction to a Work he has for several Years had on hand.

"*On the Nature of the Hepatic Function; the Purposes it serves in the Animal Œconomy; and the powerful Influence which a disordered State of it exerts, in exciting, aggravating, and modifying various Forms of Disease, both general and local.*"

In the mean time it is remarked, that public opinion has received an impulse additionally adverse to the employment of mercury.

We confess our acquaintance with the town would not have led us to this conclusion. As we before remarked, mercury seems now to have usurped the triumphal car of medicine, insomuch, as in many respects, almost to shake our former incredulity, and lead us to suspect that so general a concurrence must be founded on some experimental basis.—But of this, more hereafter.

The first pages of this "Examination" are taken up in tracing the progress of the human mind, in confirming or detecting error, and of the author in his discovery of the hepatic pathology, and the virtues of calomel. The coincidence of other practitioners is shown without any personal concurrence, and even whilst most of them are still unacquainted with the author's theory. After this follow many remarks, and not a few authorities, to show that mercury itself, given in quantities however large, and continued for any length of time, is never permanently injurious, excepting by its mismanagement. This is illustrated in a manner somewhat more trite than we should have expected from such an author, viz. That the knife and the caustic are powerful and dangerous remedies, and that the surgeon is not blamed for using a sharp knife or active caûtic.

What follows is more to the purpose, namely, that many persons who have been salivated from the mere suspicion of syphilis, have found their health permanently improved by the process. This very just remark is concluded by the introduction of a similar illustration, to which we before objected. Who, says Dr. C. would have conceived, that the amusement children derived from seeing small particles attracted by amber, would afterwards have proved to be derived from the same powers, and governed by the same laws as the destructive thunder storm!

It is again urged, that the doctrine taught by the author is confirmed by facts which have occurred to many others who have been ignorant of the principles on which those facts were to be explained. Dr. C. then admits, that there are certain forms of hepatic disease, which may be remedied by other means, yet, that there are others in which mercury is absolutely necessary.

"I should therefore," says he, "deeply lament the hasty rejection of this invaluable article; because I believe, that, if impartially estimated, it will be found to be, like small-pox inoculation, though occasionally productive of inconvenience, yet the preventive of infinitely greater mischief from the spontaneous course of the disease which it is intended to mitigate; and that until Providence shall vouchsafe to make some fortunate individual the rival of a JENNER in fame, by discovering to him (if any such exist) a perfectly harmless antidote to syphilis, and an equally efficacious remedy in hepatic complaints, we ought still to employ, with proper caution, the best in either view that we at present possess, viz. MERCURY."

We should lament as much as Dr. Curry, the hasty rejection of mercury in many chronic, and in some acute diseases; and we perfectly coincide in the quotation from Mr. Pearson, in favour of of that remedy against every form of lues venerea. Nor shall we dispute with Mr. Watt, for we have witnesed the same ourselves, that many patients, after convalescence from severe mercurial courses, were not only uninjured, but improved in their health. Still less shall we dispute the mistaken objections, at one time entertained against the Peruvian bark, or the practice of inoculation and vaccination.

We shall now offer a few remarks on some of the arguments which we stated in their order. And first, as to the negative proposition, that mercury properly administered is rarely injurious. It is obvious, that to answer such a proposition, we must first learn what is meant by the improper administration of the remedy. If the term is confined to the exposure to cold, we would answer, that under every caution of that kind, the repeated exhibition of mercury is often injurious. We have already seen how dangerous the remedy proves in the East Indies, if given when there is no other constitutional influence to oppose the actions it excites. We have seen the facts as stated by Dr. Saunders, whose experience in obstinate chronic diseases must be extensive in proportion to his standing in the profession.

Do not Pinelli and Haslam inform us, how frequently incurable madness is the consequence of repeated mercurial courses? And does not Mr. Hunter, though a warm advocate for the use of mercury, afford us many instances of the calamitous effects of its repeated exhibition. It seems incredible to us, that any practitioner, be his line in the profession what it may, has not met with pains and nodes, the offspring of mercury, and which have been exasperated by every repetition of the remedy.

It is urged, that of the present race of young men, there are few who pass the period of celibacy without the necessity of resorting to mercury, yet that few suffer materially from such a cause. That mercury may be used in such cases without permanent injury to the constitution, is not questioned by any practitioner of these days; but there are few of us, who do not recollect, how much the practice of the town is improved in this respect, and how much more frequently mercury produced permanent injury, when its exhibition was directed in such a manner as to be perpetually repeated: If men suffer less now, it is probably, because calomel is more rarely resorted to in syphilis, the crude mineral more frequently; and, if the effect produced is often considerable, the stomach and constitution are not perpetually harrassed by the mercurial salts. Dr. Curry, with his strong disposition to analogy or illustration, desires, that those who are inspired with such terrors respecting mercury, will reflect, that in some constitutions antimony will produce formidable prostration of strength, and opium watchfulness; that for each of these we have substitutes, which may answer our purpose; but that for mercury we have none. What is this but to say, that we have no medicine which excites such an action, and keeps it up so long?

It is urged that the English inhabitants of the intertropical regions are so familiarized to the use of mercury, that they dread it less than the Faculty of England formerly dreaded the Peruvian bark.—In answer to this, we need only turn to those Extracts from the Letters of Drs. Paisley and Duncan, who, though both great advocates for mercury in acute diseases, particularly those connected with the liver, are very pointed in observing the danger which attends its exhibition in many other, particularly chronic complaints.

The case of the much lamented Sir Joshua Reynolds, is instanced as one, in which the enlargement of the liver was overlooked. We confess ourselves better pleased, in this respect, with Mr. Malone's remark than with Dr. Curry's. "This instance," says the former, "may serve to show, that the patient best knows what he suffers, and that few long complain of bodily ailment without an adequate cause." Nothing can be more judiciously impressed on every practitioner, than the danger of undervaluing the complaints of his patient: but if he is always to suspect the liver, we fear the danger of such an error will be increased. Had the disease of Sir Joshua been known earlier, it is posssible it might have been remedied, but whether by calomel, is not so easily determined. Many an enlarged liver, as Dr. Saunders observes, has given way to abstinence, such exercise as the patient can bear, and occasional purgatives, which has withstood the frequent use of mercury without those auxiliaries.

It is urged by Dr. C. that we give mercury to cure a venereal ulcer and excite a considerable degree of disease. "Here," says he, "it will not be denied, that the sensible effects of the medicine, independently of its curing the ulcer, are in themselves morbid, and, *in such instances*, the utmost we can say for it is, that while we thus induce one morbid state, which is limited both in its degree and continuance, we eradicate another, the duration and mischief of which, *are known to terminate only with life.*"

We have copied this passage, just to show how extremely illogical this habit of illustration will render the most able writer. Where can be the connection between a morbid state induced in the human body and *eradications?* If a local complaint has *taken root*, it should be *dug out.* If a diseased action has taken place, it may be superseded by a different action; for this purpose, the second action must be powerful enough to supersede the former; and to be thus powerful, it must, probably, induce disease, that is, it must be greater or different from the first disease, and from the common actions of health. That mercury cures the venereal disease in this manner, was first taught by Mr. Hunter, and is now pretty generally admitted.

By parity of reason, we might expect that many long continued chronic complaints should be cured by salivation excited for a venereal ulcer, or on the suspicion that such a poison lurked in the habit. But, is this a sufficient reason for exciting a mera mercurial irritation in a vast variety of complaints, that might be relieved by ordinary means ?

It may be very justly remarked, that the venereal irritation, if not thus superseded, "only terminates with life," and that the Indian liver complaint is most urgently dangerous. In both such cases, therefore, it is more than justifiable to "induce a morbid state" of another kind. We would even admit, that slighter complaints may be superseded by an exhibition of the same remedy, the stimulus from which may not only be slight, but sometimes seem to improve, instead of injuring the health. But from experience we find the uncertainty of such an effect, and the danger of its frequent repetition, however slight.

Dr. Curry very truly remarks, that there is no remedy we can substitute for mercury; we may add, there is no other medicine, the ill effects of which, when produced to a certain degree, are so entirely unmanageable through the period of a long protracted miserable existence.

That there is nothing particularly new in this part of Dr. Curry's remarks, or in our own, we shall show by a quotation from a work published about fifteen years past, from which we extract the following passage, contained in the observations on the actions excited by mercury.*

"Hitherto we have considered only the local actions induced by mercury. But its effects on the constitution are not less deserving our attention. The fever it produces may be truly called specific, from its uniformity and total difference from all others. Hence we find it often superseding diseases kept up by habit, having arisen from causes which are no longer present. Dr. Donald Monro mentions a case of intermittent fever, which resisted all remedies, till after a mercurial salivation, it was readily cured by bark. It is not uncommon to find obstinate habitual head-aches give way to a much slighter exhibition of the same remedy : and many weak constitutions, or such as have long laboured under chronic complaints, have found, after a severe mercurial irritation, all those advantages, which often follow the energy excited during convalescence from acute diseases. These remarks are certainly foreign to our present purpose. I have, however, introduced them, because of the error some few practitioners have fallen into, of considering many diseases venereal, merely from their giving way to mercury."

By this quotation, it will appear that mercury was then in very general use on the suspicion that many chronic diseases were venereal. *Now* bilious diseases seem to have usurped the place, and in both mercury is found a valuable remedy. But are we to call all diseases

* Dr. Curry has a Note, in which he gives himself credit for much candour in not suspecting that Dr Cheyne borrowed his theory of Hydrocephalus from him. At the same time he urges, that what Dr. Cheyne published in 1808, was taught by himself in 1800, and published in his Syllabus in 1802; he therefore cannot be accused of plagiarism. When we clearly comprehend the Discovery that has been made, we will inquire with whom it originated.

diseases the effect of the venereal virus, or of disordered bilious secretions, because they yield to mercury? Or are we to resort to it in the first instance, because we so frequently find the good effects of it in cases which withstand other remedies? Is it not a fair presumption, that a remedy so efficacious must also produce injurious effects if given in constitutions very easily affected, and whilst under the impression of irritations which mercury will not supersede?

There are few things in which we should be more cautious than in overvaluing a favourite remedy, and particularly when that remedy is capable of inducing permanent mischief. Let us recollect that most diseases will, happily for the human race, cure themselves, and that when physicians are consulted, it is usually under a somewhat acute paroxysm, which is not uncommonly the natural termination of a tedious chronic complaint. We should recollect too, that those diseases usually termed nervous, are almost always, in a certain degree, relieved for a time at least, by a new remedy; and it unfortunately is not always the lot of the physician to know the future condition of his patient, though he may be in the habit of seeing him without being consulted.

If our favourite remedy is like tar water, or even antimony, one that cannot induce permanent mischief on the constitution, our error, if it is such, may be less injurious. But when we reflect, that according to the concurrent testimony of the most experienced practitioners, mercury is not to be considered in that light, it becomes us in some degree to doubt our own accuracy. None of us is ignorant that ardent spirits are a valuable remedy, that half mankind feel themselves exhilirated by such a stimulus, and that even accidental ebriety has not uncommonly proved a cure for some tedious complaints. But lest we should fall into the error of which we accuse others, in attempting to prove by illustration, we shall conclude these remarks with another quotation from the same writer to whom we last referred.

"Such is the operation of mercury on the constitution as a remedy. The constitutional diseases it produces are not less remarkable. Besides its well-known determination to the salival glands, which is neither constant nor always necessary, we find it producing head-ache, debility, and a total incapacity for application of any kind. For the most part, these gradually subside after the cause is discontinued; but in some constitutions, they end in gutta serena, epilepsy, mania, fatuity, and a train of other nervous symptoms."

On the whole, we cannot help considering the present extensive use of mercury as too indiscriminate, and by no means justified by the appearance of success it seems to be attended with. The fallacy of the latter we have already remarked, and the inconvenience of the practice we daily witness.

Of the doctrine by which Dr. Curry supports his practice, we have said little, because we can learn but little from a mere introduction. The illustration of emulging the liver by the necessity of that that practice in the mammæ, must be but a small part of what we have to learn on this subject; and from the other illustrations we have derived no information.

When the work itself appears, we promise to do it impartial justice; and if we have mistaken any thing in our preceding remarks, we shall be thankful in the mean while to be better informed.

---

### Edinburgh Journal, No. XX.

Article 1.—*A Case of Death produced by Arsenic.* By John Yellowly, M. D. Physician to the London Hospital.

The most remarkable circumstance attending this case is, that as long as the patient lived, which was twenty-four hours after swallowing the arsenic, he seemed to feel no pain. The other symptoms were such as have been usually described, viz. vomiting, purging, extreme coldness, with loss of motion in the extremities.

The appearances on dissection are worth notice. No putrefaction had taken place in the space of forty-nine hours. On viewing the cavity of the abdomen, the stomach, though very large, shewed no marks of inflammation externally, yet the whole course of the intestines, to within three inches of the *caput coli*, was much inflamed, the *duodenum* and *jejunum* were also much thickened. In some places, the external surface was florid, but mostly with occasional patches of coagulable lymph; all the lower intestines were much contracted.

On cutting into the alimentary canal, it was found, that "the inner membrane of the stomach, particularly at its lesser curvature, and in the neighbourhood of the pylorus, was very much inflamed, and there was in it frequent appearances of extravasated points of blood. In two or three circular spots, of the size of a shilling, the membrane was abraded; and, in one or two places, there was a circular thickening, (as if by an effusion of coagulable lymph), with some extravasated points of blood upon it. The stomach contained a considerable portion of a yellowish white fluid, with some castor-oil floating upon it, which had been given in the course of the day of his death. In this fluid were found a grain of shot, a few small bits of carbonaceous matter, and a very small portion of a heavy, white, coarse powder, resembling the white oxide of arsenic.

"The inflammation did not extend to the œsophagus. Portions of the small intestines were examined at several places, through their extent. The mucous membrane was every where found in a high state of inflammation, and with occasional appearances of extravasation in it. In the mucous membrane of the large intestines, the inflammation was much less, but it extended, in some degree, to the extremity of the rectum. There was no portion of arsenic or fæces found in any part of the alimentary canal."

Is this description of the inflammation extending all along the canal, canal, to be considered as the effect of arsenic having passed along it with feculent matter? or of the *continuous sympathy* of Mr. Hunter, where the adhesive inflammation cannot take place? Dr. Yellowly's remark appears very judicious, that in all such cases we should attend, not only to expelling the poisonous substance, but also to subduing the inflammation occasioned during its presence.

Article 2.—*Account of the Mortality which took place in* 1807 *among the Troops at Wallhajahad.* By a Surgeon on the Madras Establishment.

This disease was principally dysentery, which the Author attributes, besides change of climate, to the following causes. The age of the soldiers being such as to dispose them, by high health, to inflammatory diseases; exposure to the sun; transitions from heat to cold; irregularity in, or change of diet; and frequent intoxication. Yet the women and children, who could not be all exposed to these causes, were, we are told, equally victims to the disease. In some of the children, the author adds, that the influence of the sun was alone sufficient to induce it,—he does not believe the disease was, in any instance, contagious.

For the most part, the disease commenced with diarrhœa, and was seldom noticed by the patient, till he was alarmed by the appearance of blood and mucus; but sometimes the attack was more sudden, a large discharge of blood taking place whilst the soldier was on duty, or in the barracks, or even in bed. The subsequent symptoms are such as are usually met with in the worst stages of this formidable complaint.

The first intention being directed to prevention, the soldiers were confined, during the heat of the sun, to their barracks. When the disease was formed, the most successful remedies were, large doses of opium with ipecacuanha, so as to determine to the skin, which was often assisted by the warm bath. Bleeding was rarely found useful, and, what seems more remarkable, purgatives were not considered of that importance, which for some time past has been attached to them in this complaint; and mercurial frictions with calomel were only serviceable when the disease became chronic.

Article 2.—*Account of the Diseases of the Sick landed at Plymouth from Corunna.* By Richard Hooper, Member of the Royal College of Surgeons, London, late Assistant Surgeon at Old Cumberland and Mill Bay Hospital, and of his Majesty's Ship Mercury, &c.

The most important, though not the most prevailing complaint, on the return of this army, was dysentery. "This distressing disease," says our author, had, in most cases that came under my care, assumed the chronic form; the stools were very thin, frequent, and of a light yellow colour in some; whilst in others, who had laboured under this complaint for six weeks or two months previous to their arrival in this country, they were equally frequent, frequent, of a thin consistence, of a blackish dirty appearance, tinged with bloody mucous, and accompanied with a most painful tenesmus: the countenances much emaciated and dejected; their bodies and clothes dirty in the extreme, and abounding with vermin; much enfeebled, and complaining of severe pains across the loins, and in the calves of the legs; considerable thirst; little or no appetite; pulse rather frequent and small; very few had any fever, and those that had, very mild, and undeserving notice; tongue generally clean; skin rather cold, with a degree of moisture; made little or no water, some going five days successively without voiding any: these complained of a desire to make water, but, on pressing above the pubes, far from there being any tension, there was a slight depression, which evinced that the desire complained of did not proceed from a retention, but a suppression, of urine.

"The early treatment of some was five grains of calomel at night, and a smart purgative, either of natron vitriol. or pulv. jalap. the following morning; which in others was preceded by an emetic; after the operation of the purgative and emetic, small doses of the pulv. ipecac. compos. were given every four hours with a little wine; a pill, composed of calomel gr. iij. opii purificati gr. i. was also given at night. This plan of treatment, the emetics and purgatives being occasionally repeated, succeeded with those who had been slightly and recently attacked, and at first relieved the more violent cases; but after a few days these medicines produed no effect, and the disease continued with all its violence. I now changed my purgative for twenty grains of the rheum palmatum, and substituted for the pulv. ipecac. compos. a mixture, composed of pulv. aromat. gr. xij. confect. opiatæ gr. vj. spir. vini Gallici ʒiiss. mistur camphoræ ʒx. for a dose, to be repeated every four hours, the calomel and opium pill being still continued at night. The relief which this afforded exceeded my most sanguine expectations, and, on visiting my patients the next morning, all were eager in asking for a repetition of the medicine, telling me, at the same time, they had not been to stool more than six or seven times in the night; the time they were most troubled by this distressing calamity. I was induced to try the use of aromatics, from a knowledge that their powers are not entirely destroyed in the stomach, but continue through the intestinal canal, from their good effects in fistula and hæmorrhoids, hoping, by their use, to diminish the great sensibility of the intestines, and by that relieve, if not remove, the urgent tenesmus. With these views, and with the same immediate relief, I gave, to those who complained of a suppression of urine, thirty drops of the balsamum copaibæ, on lump sugar, every six hours, without the anodyne aromatic mixture. However different these medicines may be in their general nature and properties, both afforded almost instant relief, and a small quantity of urine was evacuated soon after the administration of the second dose of the balsamum copaibæ.

"Flattering

"Flattering as were the effects of these medicines, I soon found that no hopes of curing the complaint could be reasonably entertained from the use of these alone: the stools again became nearly as frequent as before, nor was their appearance changed. To the mixture I added five drops of the tinct. opii, and at each taking gave two grains of calomel, which I continued sufficiently long to produce a complete salivation in men under different circumstances, without any effect. Hitherto baffled in my attempts to relieve these miserable beings, I determined to see what a more general use of evacuants would do, and accordingly gave emetics and purgatives in alternation, continuing the use of the aromatic mixture in the intermediate time, to each dose of which was added pulv. aromatic. gr. v. tinct. opii. gtt. xv. Although this was continued for some time, it was far from encouraging, the patients being much debilitated, and the stools as before. The starch injection was next tried, to which was added forty drops of the tinct. opii; this certainly increased the frequency of the fæcal evacuations, and the patients complained bitterly of the pain the injections caused them, referring the pain to the situation of the sigmoid flexure of the colon. Being at this time informed of the benefit the sick under the care of Mr. Hewlett had derived from the use of the acetite of lead, I added 10 grains of that medicine to each injection, gradually increasing the quantity to thirty grains. This caused no more pain than the simple starch injection, nor were its operations different. Conversing with Mr. Tucker, the senior medical officer of the hospital I was attached to, on the obstinacy of this disease, he wished me to give the acetite of lead by the mouth instead of per anum, which he proposed on the principle of the action of lead in producing the opposite complaint to dysentery, the colica pictonum. I began by giving cerussæ acetatæ gr. v. confect. aromatic. q. s. ft. pilula, which was repeated every five hours: it appeared to have no effect on the constitution. My late friend and coadjutor, Mr. Williams, at the same time began by giving cerussæ acetatæ, gr. iij. opii purific. gr. iss. confect. aromatic. q. s. ft. bolus, every four hours. The frequency of the alvine discharge was much lessened on the third and fourth days from the commencement of its use, but on the fifth and subsequent days the evacuations were as before, and it was then discontinued. Having tried emetics, purgatives, the pulv. ipecac. comp. aromatics, calomel, the starch injection, acetite of lead, and opium to a certain extent, without deriving any benefit from their use, once more I thought of evacuating the alimentary canal previous to the use of opium alone. An emetic was accordingly given, which was soon followed by a purgative; after the operation of which, I began by giving two grains of opium every four hours, increasing the quantity until each dose contained six grains of the purified opium. This quantity, however enormous it may appear, was repeated every four hours for three successive days and nights, without producing the slightest change, every thing being evacuated nearly in the same state in which it was eaten."

The author now returned again to calomel and opium every night. Under this process, several were discharged cured, others had relapses, and others exchanged the complaint for hospital fever, which relieved them of all human miseries.

Typhus fever, though the most prevailing disease on the arrival of the troops, appeared at first under no very formidable shape. It is remarkable, that, contrary to general observation, the author found the disease exasperated as the weather became milder. Perhaps, however, this might have happened from the hospital being more crowded. The most alarming symptoms were confined to the head. The fourth morning generally produced a remission of the symptoms; they returned, however, by noon in full force, and continued in many till the thirteenth day, which often proved fatal. To the few who recovered, the twelfth seemed critical. Singultus, early in the disease, proved a more favourable symptom than in a more advanced period.

On the first access of the fever, vomiting and purging were directed: on the following day, antimonials with aqua ammoniæ acetatæ. On the 3d, this medicine was continued with the addition of four grs. of calomel at night. On the two following, antimonial powder with calomel gr. ij. every four hours. On the sixth, the calomel was to some increased to five gr.; and, on the following day, the colocynth was added, if necessary.

The eighth day usually commenced with symptoms of greater debility and more alarm. The remedy was changed to camphor julep with wine; the antimonial continued without calomel. On the following day the same remedies with the addition of six gr. of calomel and one of opium at night; on the following days, bark, wine, opium, and at last brandy, were given with camphor julep.

It would be injustice to the author, were we to lead the reader to suppose that these were the only remedies used. His diligence was equal to his arduous undertaking, and we wish his success had been equal to either. Cold affusions were repeatedly tried, but for the most part without any effect.. Calomel given in the beginning, and in very large doses, with colocynth on the following morning, seemed to be more successful; after this, calomel was given with antimony, as before described. If salivation was produced, the patient recovered. In some, mercurial frictions were used with calomel, and these recovered; but the surgeon, who treated them thus, died, in spite of very large doses of calomel, and frequent cold affusions. It does not appear that he used the mercurial frictions.

The appearances on dissection are too various to form any decided conclusion, as to the general cause of fatality. The appearances about the head were so natural, that instances even of congestion in the vessels of the brain were few. In many the spleen appeared an uniform mass of bloody mucus, breaking on the slightest slightest pressure, and presenting no fibrous appearance; the pancreas particularly hard, white, and much thickened. In those who died under dysentery, or typhus fever succeeding dysentery, the intestines appeared ulcerated, and sometimes gangrenous. In many, the kidneys appeared smaller and whiter than natural. These appearances are so uncertain in this viscus, that we might possibly have passed them unnoticed, had they not been coupled in the account of the inguinal glands, much enlarged and hardened.

Among all the remedies used, the author seems to think that none produced any effect excepting calomel; perhaps he is right. It is however certain that this was the only evacuant remedy tried, excepting in those cases in which it did not prove purgative. He observed also, that whenever salivation was produced, the disease seemed subdued. But does this prove any thing more than that the state of these patients was such as not to render them altogether insensible to other stimuli. That is, that the influence of the disease was not sufficient to resist the effect which would be produced by mercury on a person in health. But there is one short sentence which, whilst we admit the author's candour in introducing it, appears to us of more importance than he seems to attach to it.

"Before I conclude," says Mr. Hooper, "I think I ought to mention, that Mr. Stevenson, surgeon of the third Lancashire militia, in addition to the practice generally adopted, used large and repeated bleedings, which a reference to the proportion of deaths that occurred at the hospital under his care appears to justify."

Article 4.—*An Account of the Pseudo Syphilitic cutaneous Disease Radesyge, prevalent in some Parts of Sweden and Norway.* By H. Böcker, M. D. Upsal.

Whence this affectation of language in a science, one of the *opprobria*, of which has so often been *hard names?* Does pseudo-syphilis mean any thing? or is it only to be the title for any disease that the vulgar would call the pox, and the indolent important sagacious-looking practitioner would think he must give a name to, in order to cover his ignorance and maintain his air of superiority?

If we attend to the paper itself, we shall find no character by which we can ascertain a single disease, nor any remedy by which we can be directed in the laws it maintains from its manner of cure. At one time we find a disease like the sivvens of Scotland, spreading through a poor family by the promiscuous use, probably, of the same spoons and other utensils. Whether it is common for the poor in this country to smoak with the same pipe we are not informed. This form of the disease in the mouth and throat, we are told, is easily removed by mercury, in which it exactly agrees with the accounts of sivvens.

Many other symptoms are mentioned, as various as might be expected, when the cutaneous diseases of the poor inhabitants of a cold country are described. Some of these yield to mercury, others are exasperated by it; others relapse after being relieved by it, it, and then become incurable. Some heal of themselves in one part, whilst they break out in others; some are contagious, and others not; in short, had the author called his paper, "An imperfect Account of several cutaneous Diseases among the Poor in Sweden and Norway," he might have done some service, by inciting rational enquiry; and we might have advanced a few steps towards discriminating such as yield to mercury, from those which are likely to be exasperated, or rendered incurable by it. But the term *pseudo syphilis* serves to cover all; and the only practical inference from the paper is, to try mercury; perhaps, you will cure your patient. But suppose he should relapse? Then, try it again. But suppose he continues worse? Then try something else, or leave him to his fate.

We mean not by this to speak disrespectfully of Dr. Böeker, but to induce our readers to avoid this slovenly mode of using a general name for so many diseases, without accurately distinguishing any; and most of all, from prescribing at random, without even ascertaining by experience, the appearances which authorize us to give, or to avoid, the most important remedy with which we are acquainted. Dr. Böeker has probably not derived those advantages which Mr. Hunter's labours have afforded the British schools. Besides which, he is either ignorant of our language, or his paper has been very badly translated. We have no such English word as *humecting*, nor any such expression as *ulcers broken up*; the word *cancer* too, when connected with gonorrhœa and bubo, must mean *chancre*, which in English has its appropriate signification; *complicted*, is probably an error of the press, and is indeed of little consequence, as not likely to mislead the reader.

We shall conclude our remarks, with expressing our hopes that the author, or some other practitioner in the North of Europe, will favour us with some further and more correct information on these diseases of poverty and wretchedness.

( To be concluded in our next. )

